# The potential of exosomes in regenerative medicine and in the diagnosis and therapies of neurodegenerative diseases and cancer

**DOI:** 10.3389/fmed.2025.1539714

**Published:** 2025-03-13

**Authors:** Nikola Odehnalová, Viera Šandriková, Róbert Hromadka, Markéta Skaličková, Petr Dytrych, David Hoskovec, Zdeněk Kejík, Jan Hajduch, Frédéric Vellieux, Martina Koziar Vašáková, Pavel Martásek, Milan Jakubek

**Affiliations:** ^1^NEXARS Research and Development Center C2P s.r.o, Chlumec nad Cidlinou, Czechia; ^2^BIOCEV, First Faculty of Medicine, Charles University, Vestec, Czechia; ^3^Department of Paediatrics and Inherited Metabolic Disorders, First Faculty of Medicine, Charles University and General University Hospital, Prague, Czechia; ^4^Department of Surgery-Department of Abdominal, Thoracic Surgery and Traumatology, First Faculty of Medicine, Charles University and General University Hospital, Prague, Czechia; ^5^Department of Analytical Chemistry, University of Chemistry and Technology, Prague, Czechia; ^6^The Department of Chemistry of Natural Compounds, University of Chemistry and Technology, Prague, Czechia; ^7^Department of Respiratory Medicine, First Faculty of Medicine, Charles University and Thomayer Hospital, Prague, Czechia

**Keywords:** exosomes, regenerative medicine, biomarker, cancer, neurodegenerative diseases

## Abstract

Exosomes, nanosized extracellular vesicles released by various cell types, are intensively studied for the diagnosis and treatment of cancer and neurodegenerative diseases, and they also display high usability in regenerative medicine. Emphasizing their diagnostic potential, exosomes serve as carriers of disease-specific biomarkers, enabling non-invasive early detection and personalized medicine. The cargo loading of exosomes with therapeutic agents presents an innovative strategy for targeted drug delivery, minimizing off-target effects and optimizing therapeutic interventions. In regenerative medicine, exosomes play a crucial role in intercellular communication, facilitating tissue regeneration through the transmission of bioactive molecules. While acknowledging existing challenges in standardization and scalability, ongoing research efforts aim to refine methodologies and address regulatory considerations. In summary, this review underscores the transformative potential of exosomes in reshaping the landscape of medical interventions, with a particular emphasis on cancer, neurodegenerative diseases, and regenerative medicine.

## Graphical abstract

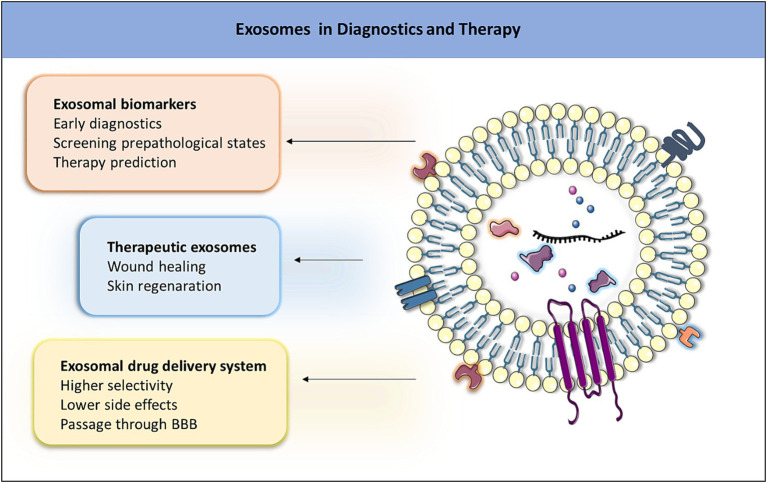

## 1 Introduction

**Figure 1:**
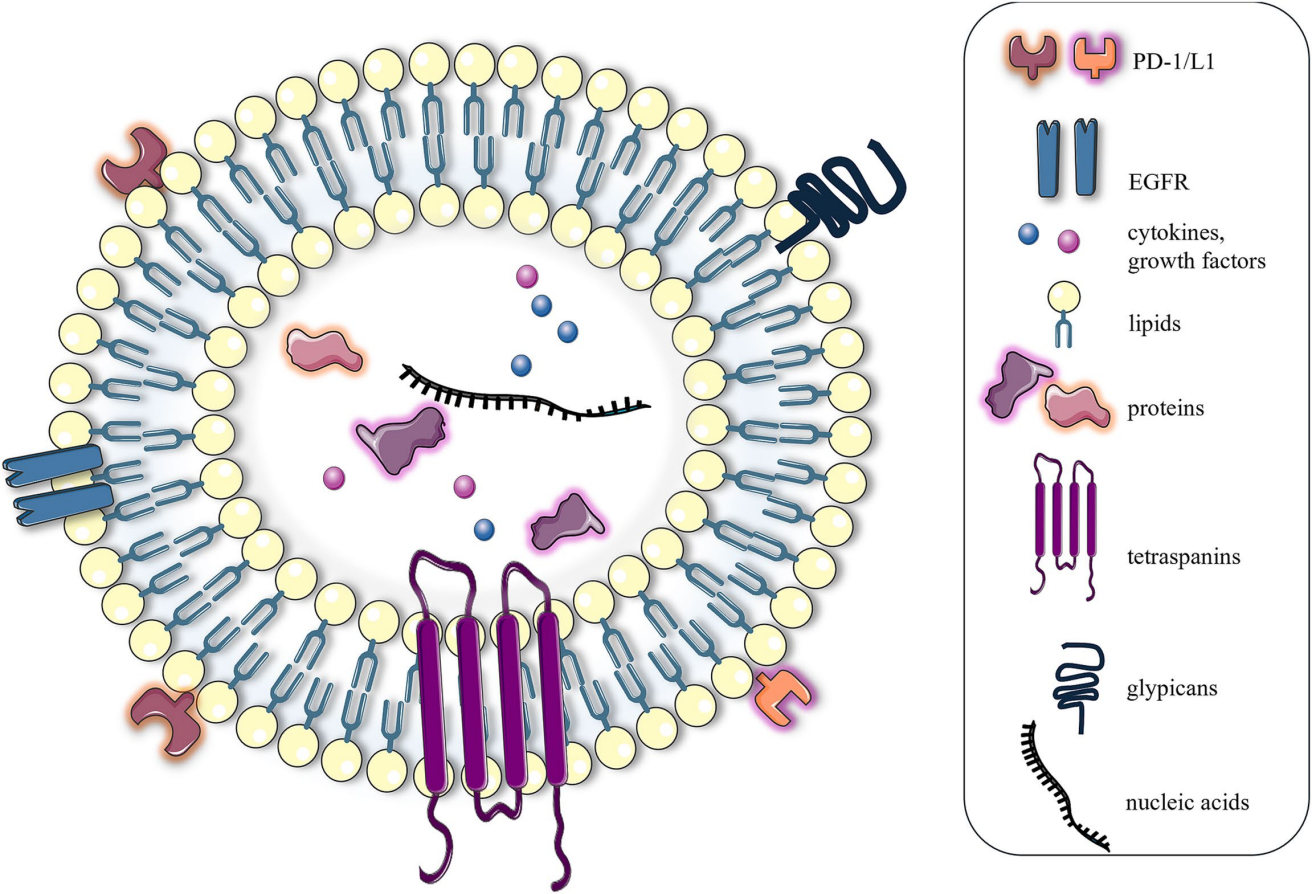
Structure of exosomes. From a structural perspective, exosomes can be defined as lipid nanoparticles characterized by a phospholipid bilayer membrane. The exosomal membrane is enriched with a diverse array of proteins and saccharide markers, including immunomodulatory molecules, such as PD-1 and PD-L1, and hormone receptors, such as EGFR, tetraspanins, and glypicans. The internal composition of exosomes comprises a variety of biomolecules, including intracellular and cytoskeletal proteins, nucleic acids, growth factors, and cytokines. The figure was partly generated using Servier Medical Art, provided by Servier, licensed under a Creative Commons Attribution 3.0 unported license.

Extracellular vehicles (EVs) are cellular structures released by cells into the extracellular space and have recently become a focal point of research due to their multifunctional role in many biological processes (1, 2). According to the new classification, EVs are divided into several types based on their biogenesis (e.g., exosome, microvesicle, apoptosome, and autophagic EVs), concept (e.g., oncosome, matrix vesicle, stress EVs, and migrasome), and size (e.g., small EVs and large EVs) (3). In general, EVs were divided into three types including apoptotic bodies (apoptosomes), which are the largest EVs with a size range between 1 and 5 μm and are released during programmed cell death (apoptosis). They contain cellular organelles and fragmented DNA and are cleared by phagocytic cells. Microvesicles, typically 100–1,000 nm in diameter, are shed directly from the plasma membrane through outward budding, a process in which a portion of the cellular membrane protrudes outward from the cell surface. They contain proteins, lipids, polysaccharides, and nucleic acids and are involved in intercellular communication and signaling (2, 3). Exosomes (Figure 1), the smallest EVs ranging in size from 30 to 150 nm, are lipid bilayer vesicles and were discovered three decades ago by Pan and Johnstone during investigations of reticulocyte maturation (2–5). Recent studies discovered small exosomes (Exo-S) and large exosomes (Exo-L). Exo-S are in the size range 40–80 nm and contain exosomal tetraspanin marker CD63, while Exo-L (80–150 nm) contain CD9 (3). Initially perceived as cellular waste products responsible for eliminating unnecessary cellular components, our understanding of exosomes has undergone a paradigm shift over the years, revealing their multifaceted functions in cellular communication and signaling (1, 2).

Exosomes not only represent a promising material for the diagnosis of serious pathological states but can also be effectively utilized for medicinal applications and drug transport. Given their significance, this review presents the medicinal potential of exosomes, encompassing areas such as regenerative medicine, early diagnosis, and drug treatment. In addition, to provide a more comprehensive understanding, the review rigorously assesses exosome biogenesis, isolation, and characterization.

## 2 Biogenesis of exosomes

**Figure 2:**
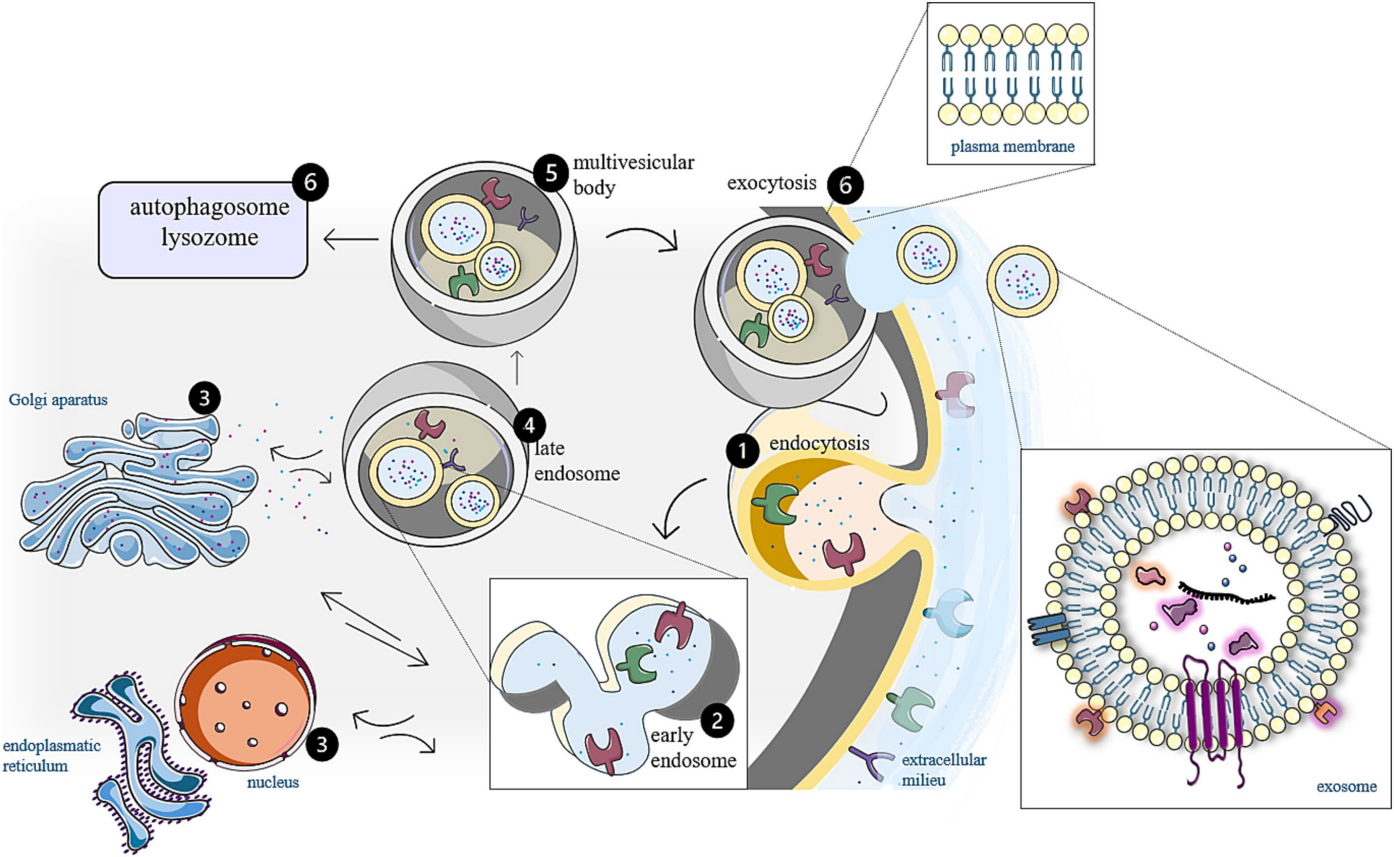
Biogenesis of exosomes. Extracellular membranes are characterized by the presence of numerous transmembrane proteins, including various receptors. (1) Upon ligand binding, receptor-mediated endocytosis is initiated, facilitated by actin filaments, which are integral components of the cytoskeleton. This process results in the invagination of the membrane surrounding the receptor, leading to the formation of an early endosome within the cell. (2) In this early endosome, the bilayer phospholipid membrane exhibits an orientation that is opposite to that of the cytoplasmic membrane, causing the extracellular domains of the transmembrane proteins to be directed inward, toward the lumen of the endosome. (3) Within the cellular context, endosomes are integral components of the complex endosomal-lysosomal system that interacts in a parallel manner (in both directions) with various organelles (e.g., Golgi apparatus and endoplasmic reticulum). This interaction allows endosomes and exosomes to encapsulate biomolecules derived from diverse cellular compartment. (4) During this process, early endosomes undergo maturation into late endosomes, which possess the normal orientation of transmembrane proteins and (5) generate intraluminal vesicles that will ultimately become exosomes. (6) Following the fusion of multi-vesicular bodies containing these intraluminal vesicles with the cytoplasmic membrane, exosomes are released into the extracellular environment. The figure was partly generated using Servier Medical Art, provided by Servier, licensed under a Creative Commons Attribution 3.0 unported license.

Understanding the biogenesis of exosomes is crucial for advancing the knowledge of their biological functions, their roles in diseases, and potential applications in therapeutics (1, 3). Their life cycle is a complex process involving three main steps: biogenesis, transport, and release (2, 3). The whole process is illustrated in Figure 2 and is initiated by an inward cell membrane budding (6). During this invagination of the plasma membrane, a portion of a cellular membrane undergoes inward folding, and a cup-shaped structure containing extracellular proteins, lipids, metabolites, and cell membrane proteins is formed. This leads to the formation of early endosomes (EEs), which subsequently mature and transform into late endosomes (LEs). Maturation involves the inward budding of the EEs membrane, leading to the sequestration of EE cytoplasmic contents and intraluminal vesicle (ILVs) formation within the endosomal lumen. During ILV formation, specific cargo molecules such as proteins, lipids, and nucleic acids are selectively sorted into the ILVs. LEs containing ILVs are called multi-vesicular bodies (MVBs). The fate of MVBs is determined by the specific proteins present on their surface, which in turn influence various intracellular pathways involved in cargo sorting and trafficking. The MVBs can either fuse with lysosomes or autophagosomes to be degraded or fuse with a plasma membrane to release the contained ILVs as exosomes (2, 6).

## 3 Isolation and characterization methods of exosomes

Isolation of exosomes is challenging due to the complexity of biological fluids (7, 8). The most common isolation methods include ultracentrifugation, ultrafiltration, size exclusion chromatography, polymer precipitation, tangential flow filtration, and immunoaffinity approaches. A comparison of these methods is provided in Table 1. The optimal isolation strategy should be selected based on the application field, as well as the volume and number of biological samples (7–9). Furthermore, the application of exosomes directly influences the use of subsequent characterization methods (9, 10). This chapter does not aim to provide a detailed explanation of the principles behind individual isolation and characterization methods of exosomes but rather to offer a comprehensive overview that assists researchers in selecting the most appropriate approaches based on considering the specific requirements of different experimental and application contexts.

**Table 1:** Advantages and disadvantages of the most common isolation techniques of exosomes.

Isolation technique	Advantages	Disadvantages	References
Ultracentrifugation	Low cost, separation of large volumes, low operating expenses, compatibility with a wide range of samples	Time consumption, low purity, inappropriateness for small volumes, diminishing biological activity of exosomes, high equipment cost	(8, 25, 26, 216, 217)
Ultrafiltration	Simplicity, fast, absence of special equipment	Potential deformation of exosomes, moderate purity, loss of exosomes, particularly problematic for isolating from small volumes, reduced purification efficiency due to clogging of membrane pores	(218–221)
Size exclusion chromatography	High purity, scalable, cost-effective for large-scale processing, availability of commercial kits, preservation of biological activity	Requirement of dedicated equipment, low yield, target product dilution	(25, 218, 220, 222, 223)
Precipitation techniques	High morphological and functional quality of exosomes, fast, simplicity, compatibility with low sample volumes, availability of commercial kits	Low purity, contamination of precipitating agent, not suitable for large sample volumes	(28, 29, 221, 224, 225)
Immunoaffinity techniques	High purity of specific exosomes	Loss of exosomes with lower expression levels, challenge separating exosomes from the bound antibodies, low capacity, low yields	(216, 226)
Tangential flow filtration	Processing of large sample volumes, high recovery rate of exosomes with minimal loss, preservation of exosome integrity, simultaneous concentration and buffer exchange (diafiltration), scalable for industrial production, reduction of processing time	High initial cost of equipment and consumables, require pre-filtration steps to remove large particles or debris, potential risk of membrane fouling leading to reduced efficiency over time	(52, 53, 55, 56)

For diagnostic purposes, exosomes from various biological fluids, including blood (serum and plasma), saliva, and urine, are used in non-invasive diagnostics due to their ability to carry specific molecular information (9, 11). Blood-derived exosomes provide systemic insights, reflecting the physiological and pathological state of the entire body, making them ideal for detecting various diseases such as cancer, neurodegenerative, cardiovascular, and autoimmune diseases (12–16). Salivary exosomes, primarily originating from the salivary glands and the oral cavity, are valuable for diagnosing oral diseases and gastrointestinal tract disorders (17–19). Urinary exosomes, secreted by epithelial cells of the urinary tract, are effective in diagnosing renal diseases, bladder cancer, and prostate conditions (20–22).

When utilizing exosomes for diagnostic purposes, the primary goal is to identify disease-specific exosomal markers. To determine disease-specific markers, it is essential to identify markers that differ in presence or expression level between samples from diseased and healthy patients. This requires the comparison and processing of a large number (tens to hundreds) of biological samples for statistical relevance (23, 24). This procedure thus involves processing large numbers of samples with small volume, typically in the maximum of a few milliliters. For limited numbers of samples, typically in the range of lower tens, differential ultracentrifugation (DUC) is considered the gold standard and is the most widely used method. It usually involves several consecutive rounds of centrifugation with increasing centrifugal force and centrifugation time to remove cells, cell debris, and larger microvesicles. The final step at 100,000 g or higher serves to precipitate the exosomes (12, 25–27).

Because DUC is time-consuming, for handling tens to hundreds of samples, the final ultracentrifugation step is often replaced with commercial kits based on precipitation. Several commercial kits use a polyethylene glycol precipitation technology, including the Total Exosome Isolation Kit (Invitrogen), ExoQuick-TC Exosome Precipitation Solution (System Biosciences), miRCURY Exosome Kits (QIAGEN), Exo-Prep (HansaBioMed), PureExo Exosome Isolation Kit (101Bio), ExoGAG (NasasBiotech), Exosome Precipitation Solutions (Immunostep), and the miRCURY Exosome Isolation Kit (Exiqon) (28–32). These methods result in the isolation of exosomes referred to as total exosomes. In neurodegenerative diseases (NDs), specific subpopulations of exosomes are isolated from the total exosome pool using immunoaffinity methods with specific antibodies, such as anti-L1CAM, anti-NCAM, anti-MOG, or anti-GLAST. These antibodies bind specifically neural, oligodendrocyte, or astrocyte exosomes, which are the most relevant for identifying markers of NDs as Alzheimer disease (AD), multiple sclerosis (MS), dementia, or schizophrenia (33–36).

After the isolation of total exosomes or specific exosomal subpopulations, the characterization of these exosomes and the identification of specific disease markers are performed (10). The most common markers include specific proteins or RNAs. For protein marker identification, techniques such as tandem of liquid chromatography and mass spectrometry (LC–MS) are used, while next-generation sequencing (NGS) is used for RNA. Once specific disease markers are identified, it is crucial to determine whether their exosomal expression differs significantly between diseased and healthy patients in a statistically relevant manner. To quantify markers, techniques such as enzyme-linked immunosorbent assay (ELISA) or quantitative reverse transcription polymerase chain reaction (qRT-PCR) are commonly used (37–42).

Currently, exosomes are being extensively investigated as drug delivery systems. Due to their nanoscale dimensions, they can deliver therapeutic agents specifically to tumor sites (43). Tumor tissues are characterized by a high absorption capacity and poor drainage (44), which facilitates the selective accumulation of exosomes with prolonged circulation times within these tissues. In addition, it is noteworthy that exosomes exhibit favorable permeability across BBB (45). Furthermore, the surface of exosomes can be effectively modified with various ligands that are specific to target cells, thereby significantly enhancing the selectivity of exosomes for these cells (46). As cell-derived products, exosomes are generally considered to be safer than conventional nanoparticles, particularly metal-based nanoparticles (47). Their unique structure, comprising a hydrophilic core and a lipid bilayer, allows them to transport a wide range of drug types (46). However, a significant limitation in the clinical application of exosomes is that cells produce only small quantities of various types of exosomes, which restricts their usability in therapeutic contexts (48).

In regenerative medicine and therapeutic applications, exosomes are isolated from cell culture media. The most commonly used cell sources are mesenchymal stem cells. Similar to diagnostic applications, where DUC is a preferred method for smaller sample quantities, DUC is also commonly employed for isolating exosomes from cell culture media for volumes ranging from tens to hundreds of milliliters. However, a drawback of the final ultracentrifugation step is that the high speeds lead to exosomal damage and the sedimentation of impurities, which diminishes exosomal therapeutic activity (12, 49, 50). Therefore, the final ultracentrifugation step is often replaced by density gradient ultracentrifugation (DGUC), utilizing sucrose or iodixanol gradients (OptiPrep™), where the exosomal fraction is not only less damaged but also better purified (49–51).

For larger volumes, in the range of hundreds of milliliters to liters, a combination of other techniques is employed due to the limited capacity of centrifuges. These primarily include ultrafiltration (UF), utilizing polymer filters of various pore sizes to remove cells, cell debris, and microvesicles, followed by tangential flow filtration (TFF) to remove contaminating proteins, to concentrate the sample, and to perform diafiltration of exosomes into the desired buffer (52–54). For final exosome purification, size exclusion chromatography (SEC) is employed, followed by a final TFF step to concentrate the sample and transfer it into suitable application buffers, most commonly PBS (55–57). The advantage of the combination of techniques such as UF, TFF, and SEC is that they are suitable for practical use on an industrial scale (55, 56). In addition, the isolated exosomes exhibit high purity and preserved therapeutic activity, which is usually subsequently confirmed by *in vitro* tests such as scratch or transwell assays (54, 58).

According to the recommendations of the International Society for Extracellular Vesicles (ISEV), exosomal samples should generally be characterized for size, concentration, and the presence of exosomes (59, 60). The presence of exosomes is typically confirmed by detecting at least one transmembrane protein (commonly tetraspanins: CD9, CD63, and CD81) or a GPI-anchored protein (e.g., integrins), along with one cytoplasmic lipid (e.g., sphingolipids, ceramides, and cholesterol) or cytoplasmic protein (e.g., ALIX, TSG101, and HSP70) (59, 60). These specific markers can be assayed using methods such as Western blot or ELISA (61, 62). In addition, electron microscopy is often employed to provide images of typical exosomal morphology, further confirming the presence of exosomes in the sample (63, 64).

The size and concentration of exosomes, expressed as particles per milliliter, are commonly determined using techniques such as dynamic light scattering (DLS), nanoparticle tracking analysis (NTA), and resistive pulse sensing (RPS). However, these techniques are not specific to exosomes and may overestimate their concentration (65, 66). Alternatively, exosomes can be labeled with specific dyes, where the measured concentration corresponds only to the positively stained population. This approach allows for more accurate quantification of exosomes and can be performed using nanoflow cytometry (nanoFCM). Nevertheless, underestimation of concentration may occur if the sample is not properly titrated (67, 68). In some cases, the concentration of exosomes is determined based on the total protein concentration per milliliter (μg/mL) using the Bradford assay (10). All these parameters must be thoroughly assessed to ensure sufficient sample purity and demonstrate that the observed therapeutic effect is primarily induced by exosomes rather than by potential contaminants.

## 4 Therapeutic applications of exosomes in biomedicine

**Figure 3:**
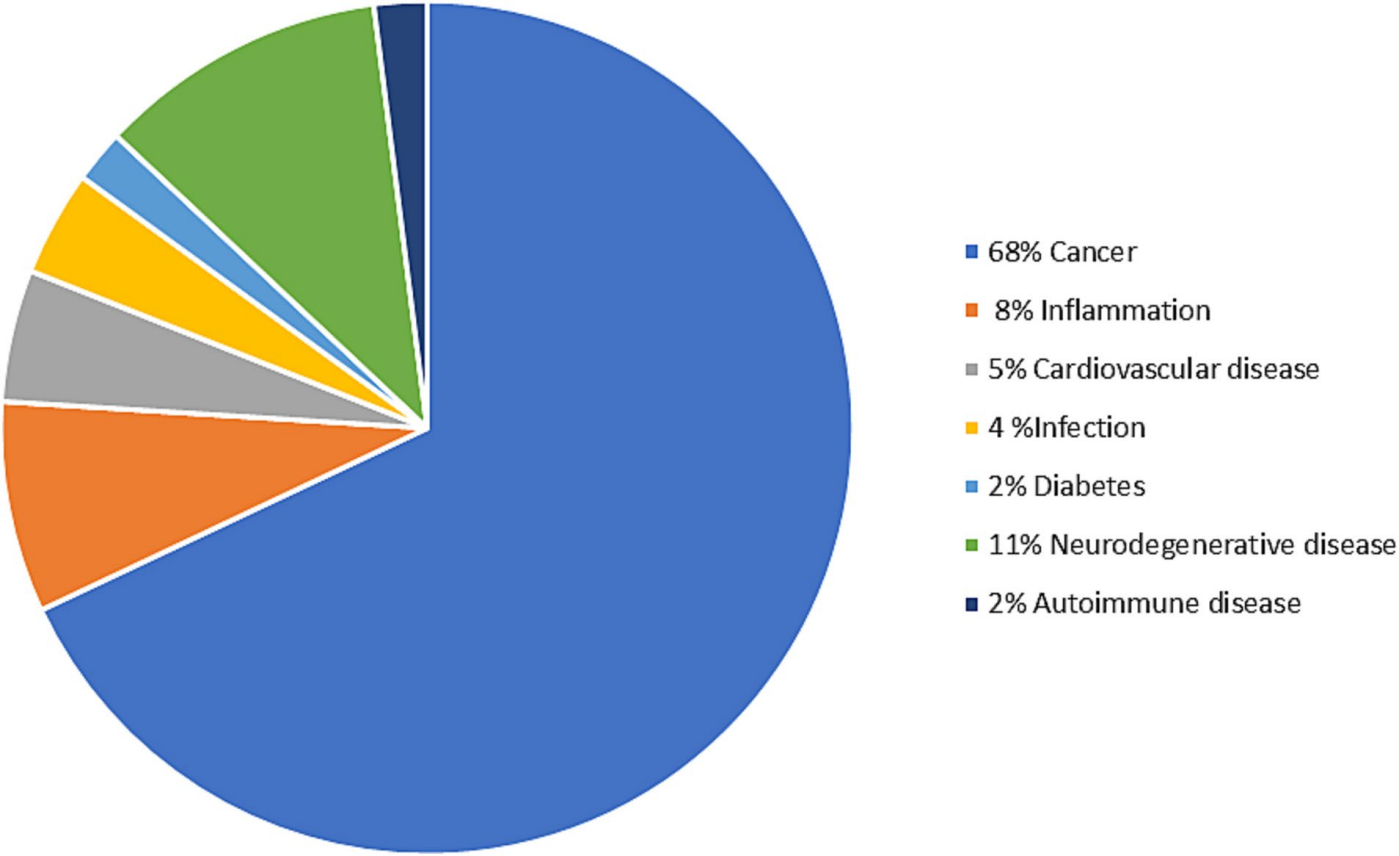
Distribution of exosome therapy and diagnostics concerning the target diseases (74). The figure was partly generated using Servier Medical Art, provided by Servier, licensed under a Creative Commons Attribution 3.0 unported license.

As mentioned earlier, exosomes are natural intercellular communicators in normal biological processes but also in pathologies. They transport proteins, lipids, and nucleic acids specific for their parenteral pathogenic cells. From a clinical perspective, most applications use exosomes as biomarkers of diseases (69). The content of the exosome has been shown to be disease-specific, such as in NDs, prion diseases, viral infections, and cancer (69). Furthermore, exosomes play a transformative role in regenerative medicine, offering innovative therapeutic interventions. Their bioactive cargo, including growth factors and signaling molecules, has demonstrated significant potential in modulating immune responses and promoting tissue repair (70). In addition, exosomes serve as carriers for therapeutic cargo loading, holding promise for targeted drug delivery in disease treatment (69, 71, 72). Currently, 150 clinical trials registered on ClinicalTrials.gov are investigating exosome-based therapies for various diseases (73). The majority of applications utilizing exosomes for both therapy and diagnosis focus on their utilization in the fields of cancer and NDs, as depicted in Figure 3 (74). For this reason, this review will focus on the use of exosomes in these diseases.

### 4.1 Exosomes in regenerative medicine

Nowadays, one of the main applications of exosomes is in regenerative medicine, a promising field dedicated to the regeneration and reconstruction of diseased or injured organs and tissues (72). Since the 1970s, mesenchymal stem cells (MSCs) have been under investigation in this field for their multipotent characteristics and their ability to migrate to injury sites. They are used in regenerative medicine due to their robust self-renewal capacity and ability to differentiate into adipogenic, chondrogenic, osteogenic, endothelial, neural, and epithelial cells, as proven in both *in vivo* and *in vitro* experiments (70, 73–75). MSCs, like every cell in the human body, release exosomes, which have started to be extensively researched due to their regenerative properties. Currently, at least 31 clinical trials are exploring the use of exosomes derived from MSCs (MSCs-EXOs) as an alternative to basic MSCs therapy (73). MSCs-EXOs have shown comparable or superior therapeutic efficacy compared to MSCs alone (72, 73). They exhibit lower immunogenicity, present an enhanced safety profile by avoiding concerns related to uncontrolled differentiation and tumorigenicity associated with live cells, and offer improved storage conditions, simplifying logistical challenges compared to live cell storage. In addition, there are no ethical issues, and their small size allows for sterilization by filtration (73–77). The comparison between exosomal and stem cell therapy is provided in Table 2.

**Table 2:** Exosomes versus stem cells in preparation and therapy.

	Advantages	Disadvantages
MSC preparation	Ease to isolate, easy to expand at a large scale, highly proliferative, well established FDA guidelines	Harsh storage and transportation conditions
Exosome preparation	Small size, stable upon freezing and thawing	Difficult to isolate and purify, no established regulations and standards
Therapeutic application of MSCs	Multilineage differentiation potential, extensive preclinical and clinical studies	Immunogenicity, oncological complications, fusion toxicity, ethical issues
Therapeutic application of exosomes	Minimal risk of immune responses and tumor formation, no ethical issues, multiple delivery routes, can be engineered to specifically target and deliver drug cargoes	Rapid clearance from blood after administration

The greatest attention in regenerative medicine is focused on skin healing, a tissue which plays a crucial multifunctional role as a protective barrier, a temperature regulator, and facilitating tactile and pain sensations (78). However, the use of exosomes for wound treatment could be challenging due to their rapid clearance from the application site, which limited therapeutic effectiveness (79). To address this limitation, current research focuses on the combination of exosomes and biomaterials. This innovative approach extends the retention time of exosomes on the wound surface without compromising their biological activity, enabling the development of advanced exosome-based therapies (80, 81). Hydrogels as biomaterials exhibit a synergistic effect in exosome-induced wound healing and can serve as a versatile platform for the incorporation of therapeutic exosomes, enhancing their efficacy in tissue regeneration. To date, hyaluronic acid, gelatin, chitosan, and polypeptide-based hydrogels have been used for encapsulating exosomes from different cell sources (75, 80, 81). Chitosan is the often-used material for hydrogel preparation. Chitosan hydrogel enriched with MSCs-EXOs, specifically human endometrial stem cell-derived and human placenta-derived exosomes, demonstrated notable wound closure ability by promoting the formation of new epithelial cells, significant retention of MSCs-EXOs at injury sites, promotion of angiogenesis, and acceleration of the recovery of ischemic hind limbs (82). For the best contact of the hydrogel with the wounded skin and effective wound filling, thermosensitive hydrogels are used. Thermosensitive pluronic hydrogel combined with human umbilical cord MSCs-EXOs (HUCMSCs-EXOs) significantly accelerated wound closure, promoted angiogenesis, and improved skin healing of chronic diabetic wounds (83). For effective diabetic wound treatment, polyvinyl alcohol/alginate nanohydrogel with HUCMSCs-EXOs and an injectable antibacterial polypeptide-based hydrogel with adipose derived MSCs-EXOs were used. This type of hydrogels demonstrated the ability to promote proliferation, migration, and angiogenesis of human umbilical vein endothelial cells, expediting diabetic wound closure thus presenting a novel approach for complete skin regeneration (84, 85).

Further applications in regenerative medicine are focused on hard tissue regeneration, essential for bone and cartilage repair. Traditionally, MSCs, scaffolds, and growth factors are used in this field. While scaffolds have been proven beneficial for bone regeneration, the avascular nature of cartilage poses unique challenges (75). Osteoarthritis (OA), a prevalent joint disease extending beyond cartilage, demands innovative regenerative procedures (81). Promisingly, exosome-integrated scaffolds and MSCs-EXO therapy show potential in OA treatment. Articular cavity injection with HUCMSCs-EXOs in PBS demonstrated significant efficacy in preventing severe damage to knee articular cartilage in a rat OA model. These therapies not only promoted chondrocyte proliferation and migration but also exhibited anti-apoptotic effects and reversed cellular injuries. Moreover, HUCMSCs-EXOS played a crucial role in regulating the polarization of M2 macrophages, fostering chondrocyte survival by producing anti-inflammatory cytokines to suppress adverse inflammation (86). To enhance therapeutic efficiency and retention time *in vivo*, HUCMSCs-EXOs were engineered to specifically target chondrocytes and encapsulated within hyaluronic acid hydrogel, presenting a “two-phase” releasing system. This approach synergistically facilitated OA cartilage repair in a rat model and proved the rejuvenating effects of HUCMSCs-EXOs on aging chondrocytes in OA, offering a promising cell-free OA treatment strategy (87). Therapeutic potential of bone marrow MSCs-EXOs was explored in the context of mitochondrial dysfunction and oxidative stress in OA. 3D printed scaffolds, composed of extracellular matrix, gelatin methacrylate, and exosomes, effectively restored chondrocyte mitochondrial function, enhanced chondrocyte migration, and polarized the synovial macrophage response *in vitro*. Notably, a 3D printed scaffold significantly facilitated cartilage regeneration in a rabbit model, highlighting its potential as an early treatment strategy for OA (88).

In recent years, MSCs-EXOs have also been used increasingly in ophthalmology. MSCs-EXOs have shown promise in various applications, such as promoting ocular tissue regeneration and addressing vision-related disorders. MSCs-EXOs have explored the therapeutic potential of stem cell exosomes in treating ocular surface diseases, corneal injuries, and retinal degenerative conditions (89). Exosomes derived from bone marrow stem cells have demonstrated the ability to enhance corneal epithelialization and maintain corneal transparency in diabetic mice (90). MSCs-EXOs have been explored for their regenerative effects on corneal injuries (91). In retinal diseases, including age-related macular degeneration and retinitis pigmentosa, MSCs-EXOs have been investigated for their neuroprotective and regenerative properties. These exosomes may influence retinal cell survival, angiogenesis, and anti-inflammatory responses, offering a novel approach for treating degenerative conditions affecting the retina (92). As exosome research in regenerative medicine continues to progress, MSCs-EXOs are beginning to be applied into various fields, such as periodontitis (93, 94). Research on exosomes in regenerative medicine is expected to gain prominence, highlighting the growing need to integrate exosome-based regenerative therapies into clinical practice (95).

### 4.2 Exosomes in early diagnosis

**Figure 4:**
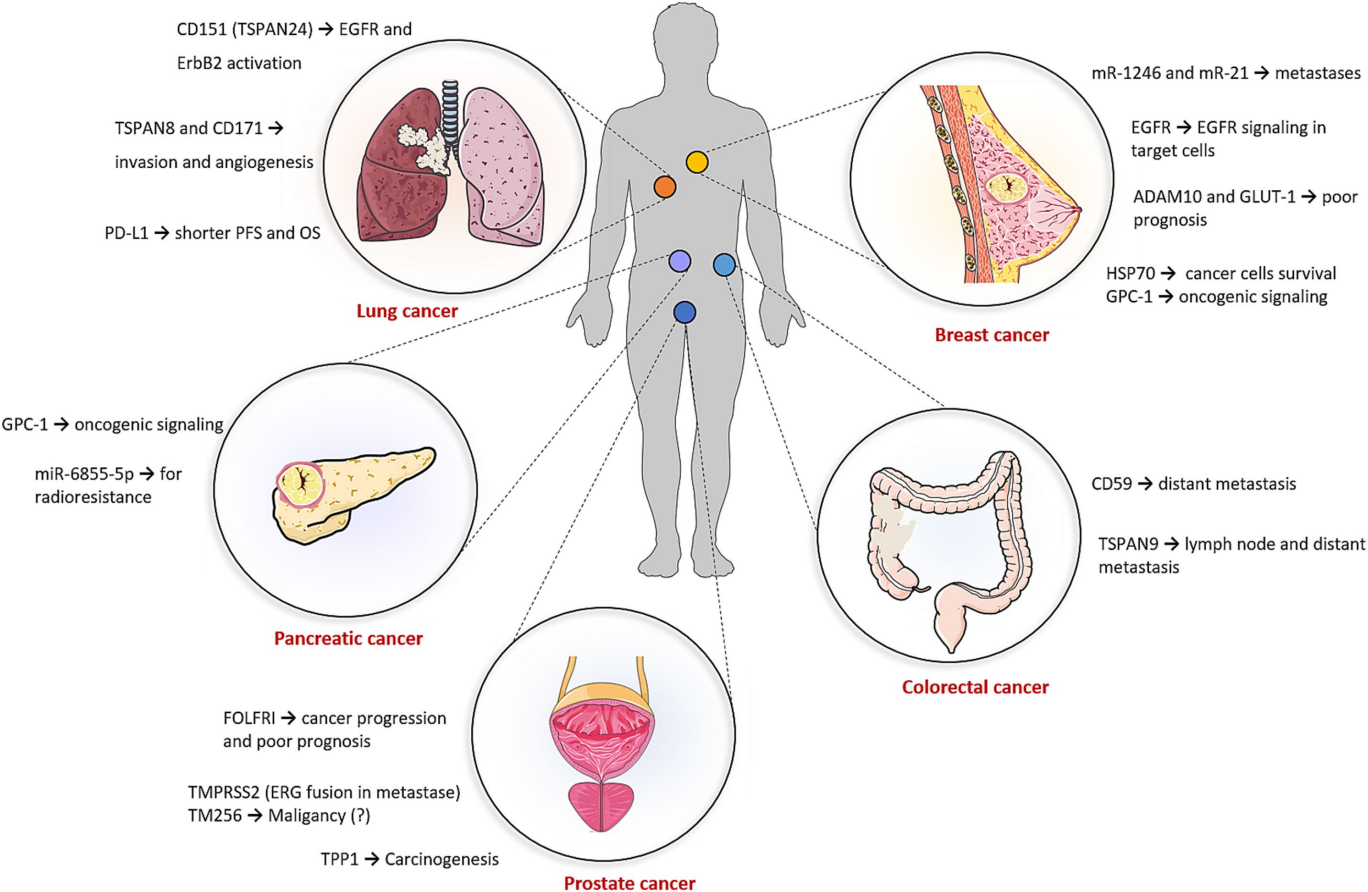
Potential of exosomes in cancer diagnostics. The applicability of exosomes in cancer diagnostics is detailed in Supplementary Table S1. The figure was partly generated using Servier Medical Art, provided by Servier, licensed under a Creative Commons Attribution 3.0 unported license.

As already mentioned, exosomes are promising biomarkers for the diagnosis of several diseases. Most attention has been paid to cancer (Figure 4; Supplementary Table S1) (95–105) with less focus on NDs.

Tumor-derived exosomes (TEXs) have emerged as critical players in cancer progression. Cancer cells release TEXs in large quantities, leading to a rapid increase in the concentration of total exosomes in the serum or plasma of cancer patients, which correlates with poor prognosis (106). TEXs provide immunosuppressive effects by inducing dysfunction in various immune cells, thereby suppressing anti-tumor immune responses (107). Initial interactions between TEXs and immune cells occur through ligand–receptor recognition, followed by either direct fusion with the plasma membrane or receptor-mediated uptake, resulting in the release of TEX cargo into the cytoplasm of immune cells. While T lymphocytes do not efficiently internalize TEXs, they interact with surface molecules to trigger sustained Ca^2+^ flux and downstream signaling cascades, ultimately altering the transcriptome of recipient cells. In contrast, phagocytic cells, such as dendritic cells and macrophages, rapidly internalize TEXs (108).

Macrophages are a prominent component of the tumor microenvironment (TME) and can make up more than half of the tumor mass in some cases. Moreover, tumor progression is associated mainly with macrophages. Macrophages can be induced to either tumor-suppressive immunological type M1 or tumor-promoting inflammatory M2 macrophages (109, 110). Tumor-associated macrophages predominantly exhibit an M2 phenotype, characterized by the secretion of pro-angiogenic factors and cytokines that promote angiogenesis, tumor growth, and metastasis (111, 112). Many studies have shown evidence on the role of TEXs in macrophage M2 polarization to promote tumor progression. For instance, exposure of macrophages to TEXs decreased the expression of M1 markers, such as IFNγ, while upregulating IL-1β, a marker of inflammation, suggesting that TEXs may help maintain a macrophage phenotype supportive of tumor survival and proliferation (113). In triple-negative breast cancer (TNBC), TEXs have been reported to promote M2 macrophage polarization, facilitating lymph node metastasis. Co-culture of TNBC-TEXs with macrophages led to significant morphological changes and an increased expression of M2 markers, including Fizz1, CD206, and Arg-1. An orthotopic TNBC model further confirmed the role of TEXs in driving M2 polarization, with enhanced tumor growth and axillary lymph node metastasis observed *in vivo* (114).

In cancer metastasis, epithelial–mesenchymal transition (EMT) is the key process during which cancer cells lose their epithelial properties and adopt a more mesenchymal and invasive phenotype. Generally, EMT initiation is characterized by loss of cell–cell adhesions and apicobasal polarity, leading to the formation of cells with increased migratory and invasion capabilities that are able to invade the extracellular matrix (115, 116). At a molecular level, EMT involves the downregulation of epithelial-type proteins, such as E-cadherin, and the acquisition of mesenchymal markers, such as vimentin (117). TEXs from bladder cancer cells have been shown to induce EMT-like changes in urothelial cells, enhancing their invasive potential. This effect was mediated by TEX-induced upregulation of vimentin and downregulation of E-cadherin through the TGF-β1 signaling pathway (118). Similar EMT-inducing effects of TEXs have been observed in glioblastoma, lung carcinoma, and gastric cancer models (119–121). TEXs can also influence EMT through their miRNA cargo. For example, miR-23a within TEXs promotes EMT by inhibiting E-cadherin synthesis in lung carcinoma and melanoma cells, while miR-191 and let-7a, present in TEXs from patients with melanoma, gastric, and colorectal cancers, have also been implicated in EMT regulation (122–126). In addition, TEX-derived miR-105 has been shown to promote vascular invasion by downregulating ZO-1 in endothelial cells. Notably, elevated miR-105 levels in the serum of breast cancer patients have been correlated with metastatic progression and poor prognosis (127). Given their critical role in modulating the TME, promoting EMT, and facilitating immune evasion, profiling TEXs in blood and other body fluids holds significant promise as a non-invasive method for cancer diagnosis and prognosis (128).

The WHO Global Cancer Observatory (GLOBOCAN) 2022 registry provides a list of the most common types of cancers. The three most common types are lung (12.4%), breast (11.5%), and colorectal (9.6%) cancers. Lung cancer is the leading cause of cancer death worldwide (129). Surface-enhanced Raman spectroscopy (SERS) of exosomes, combined with AI deep learning software, allows for accurate diagnosis of early-stage lung cancer. The deep learning model was trained with SERS signals of exosomes derived from normal and lung cancer cell lines and subsequently its ability to detect cancer was verified using exosome samples from patients’ blood. The model identified the lung cancer patients and even detected stage I patients with an accuracy of 90.7% (130). Regarding TEX protein biomarkers, CD151, TSPAN8, and CD171 were overexpressed in lung cancer samples. Of these, CD171 has been associated with EMT, metastases, and poor prognosis (96, 131, 132). Determination of PD-L1 can also provide important diagnostic information. Akbar et al. reported that exoPD-L1 from non-small cell lung cancer (NSCLC) patients and healthy controls showed a significantly higher difference than corresponding serum and tissue PD-L1 (97). For example, exoPD-L1 was found in all the patients (100%), while tissue PD-L1 was observed only in 71% patients. Furthermore, exoPD-L1 can also be used for the prediction of immune inhibitors efficiency. ROC curve analysis of change from baseline in exoPD-L1 levels between responders and non-responders showed 87% sensitivity and 100% specificity (*p* = 0.0015), indicating strong discriminatory power. An increasing number of studies have demonstrated that non-coding RNAs are closely correlated with the initiation and development of lung cancer as well (133). Detection of exosomal long non-coding RNA as potential lung cancer diagnosis is also performed in a current clinical study (NCT03830619) (134). Breast cancer is the most common type of cancer and the second leading cause of cancer-related deaths in women. In this type of cancer, media from breast cancer cell lines were analyzed to identify specific exosomal proteins such as glucose transporter-1, glypican-1, and the metalloproteinase domain-containing protein 10 (98). Epidermal growth factor receptor (EGFR) is a transmembrane protein that plays a key role in cell signaling pathways involved in cell growth, proliferation, and survival and is often overexpressed or mutated in various cancers. It was reported that triple-negative breast cancer cells (MDA-MB-468) can produce exosomes with encapsulated EGFR (protected from EGFR inhibitor), which can induce EGFR signaling in target cells, thereby promoting cancer progression or resistance to therapy (99). Typical non-coding RNA cancer biomarkers are miR-1246 and miR-21, which were also overexpressed in breast cancer cell lines (100). The plasma level of miR-1246 was measured in breast cancer patients and healthy controls using an Au nanoflare probe. This biomarker-based probe distinguished breast cancer patients from healthy individuals with 100% sensitivity and 92.9% specificity (95). Currently, a clinical trial (NCT02662621) is underway, focusing on an exosomal detection protocol for diagnosing various cancers, including breast cancer. The study indicates that exosomes displaying the stress protein HSP70 on their membrane may serve as cancer-specific exosomes (134). In colorectal cancer, tetraspanin-1 was found to be upregulated in plasma exosomes from patients compared to healthy controls, showing 75.7% sensitivity (135). Pancreatic cancer, one of the deadliest cancer types, involves TEXs that carry the glypican-1 biomarker. This co-receptor for various signaling molecules regulates key processes such as cell growth, motility, and differentiation (102). Currently, two clinical trials are underway to assess the efficacy of diagnostic exosomes in colon and liver cancers (NCT03432806) and in pancreatic cancer (NCT03334708). These studies aim to identify biomarkers circulating in blood and analyze the corresponding tissues (134). While previous studies have focused on TEXs from blood, urinary exosomes play a role in urological tumors (136). In prostate cancer, biomarkers such as TM256 and LAMTOR proteins found in urinary exosomes exhibited very high sensitivity (105). In addition, potential biomarkers such as TPP1, TMPRSS2, and FOLR1 were highly upregulated in urinary exosomes derived from the bladder. Notably, despite being histologically tumor-free at cystectomy, patients’ urinary exosomes displayed a carcinogenic metabolic profile, likely originating from undetected or partially transformed cancer cells (137).

**Figure 5:**
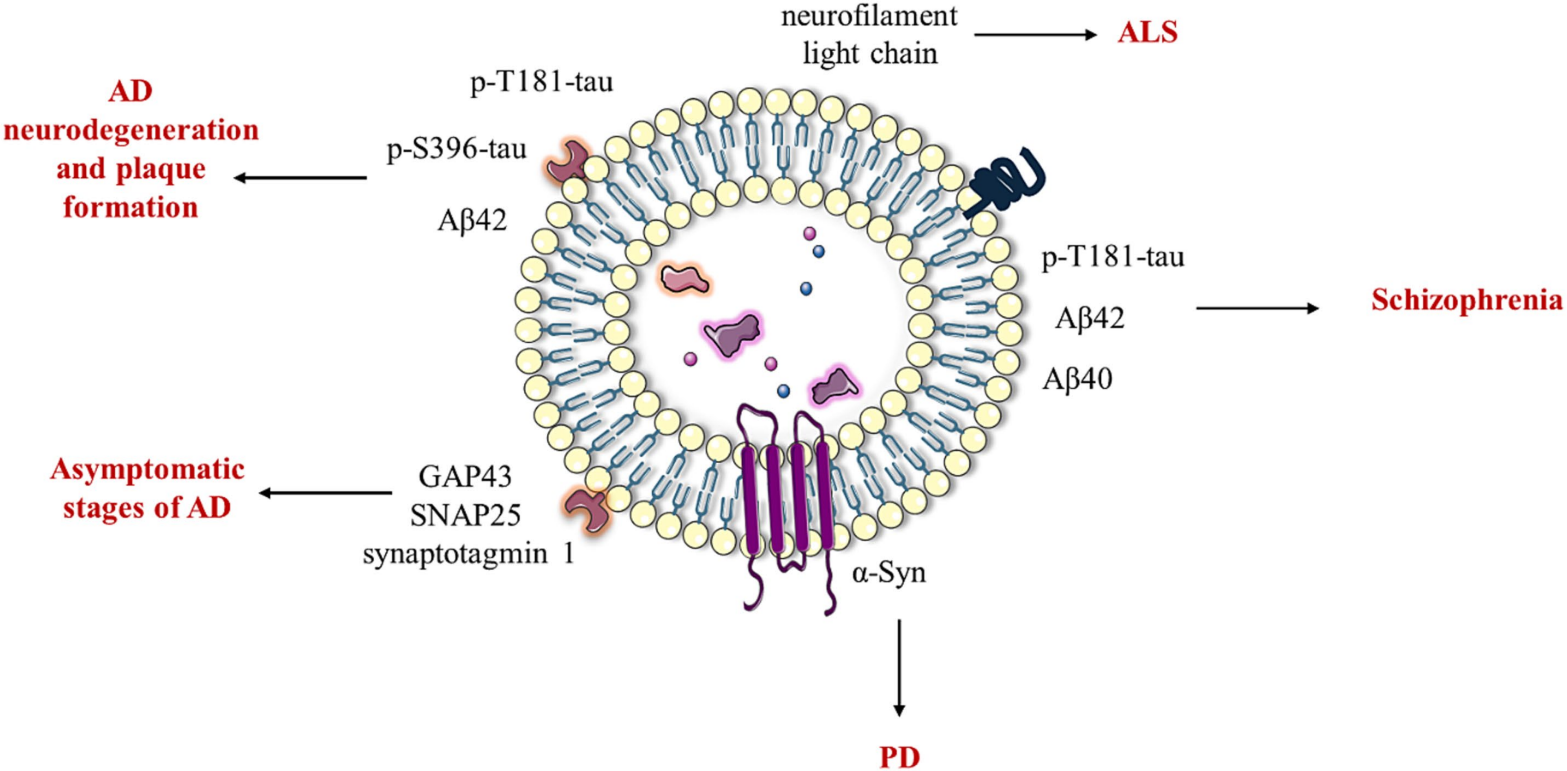
Potential of exosomes in the diagnosis of neurological diseases. In AD, protein markers such as total tau, phosphorylated T181-tau (p-T181-tau), phosphorylated S396-tau (p-S396-tau), and amyloid-beta 42 (Aβ42) derived from neural-derived blood exosomes are indicative of neurodegeneration and plaque formation. Notably, GAP43, SNAP25, and synaptotagmin 1 can be detected even in asymptomatic stages of AD. Furthermore, exosomal Aβ42, Aβ40, and P-T181-tau have been implicated in schizophrenia. In the context of PD and ALS, exosomal α-synuclein and neurofilament light chain exhibit promising potential as biomarkers for disease monitoring and progression. The figure was partly generated using Servier Medical Art, provided by Servier, licensed under a Creative Commons Attribution 3.0 unported license.

Current diagnosis of NDs relies on clinical assessments, medical history, imaging techniques, and diagnostic tests based on observed symptoms (138, 139). However, predicting these diseases remains challenging due to the typically late-stage diagnosis. Emerging research highlights the potential of exosomes as biomarkers for early detection, offering a promising approach for more effective and timely diagnoses in the future (Figure 5) (140). Early diagnosis of NDs is crucial for enabling timely interventions that can significantly improve patient outcomes (141).

In Alzheimer’s disease (AD), neural-derived blood exosomes have emerged as a valuable source of AD-related overexpressed protein markers, including total tau, P-T181-tau, P-S396-tau, and Aβ42 (33). Exosomal synaptic proteins, such as growth-associated GAP43, neurogranin, SNAP25, and synaptotagmin 1, show promise in predicting AD at asymptomatic stages, potentially detecting the disease 5 to 7 years before cognitive impairment occurs (34). In addition, miRNA markers, including miR-137, miR-181c, miR-9, and miR-29a/b, are downregulated in blood serum and offer additional potential for early AD detection (142, 143). Among other types of biomarkers, lipids such as LDL-C, TG, and HDL-C are associated with AD (144). Furthermore, it was also shown that exosomal protein markers offer comparable diagnostic capacity to cerebrospinal markers, further enhancing the potential of blood-based diagnostics for AD (145). Exosomal levels of Aβ42, Aβ40, and P-T181-tau were also measured in the blood plasma of patients diagnosed with schizophrenia (36). Specifically, these markers were determined in neural and astrocytic exosomes. While Aβ42 levels were higher in astrocytic exosomes than in neural exosomes, other markers were similar between these two groups. It was also found that higher astrocytic P-T181-tau levels were associated with worse executive functioning, and astrocytic Aβ42 levels were more sensitive and specific in differentiating diagnostic groups. Other types of neural cells explored in the context of NDs are oligodendrocytes. These cells are associated with MS. Oligodendrocyte-derived extracellular vesicles showed higher concentrations of myelin basic protein, which could serve as potential biomarkers across diverse MS phenotypes (35). For Parkinson disease (PD), it was found that the most reliable biomarker of blood neural exosomes is *α*-synuclein (146). In amyotrophic lateral sclerosis (ALS), exosomal biomarkers, such as neurofilament light chain, have been identified in both cerebrospinal fluid and blood. This marker can serve for early diagnosis and monitoring disease progression (147).

### 4.3 Exosomes in targeted delivery and disease treatment

**Figure 6:**
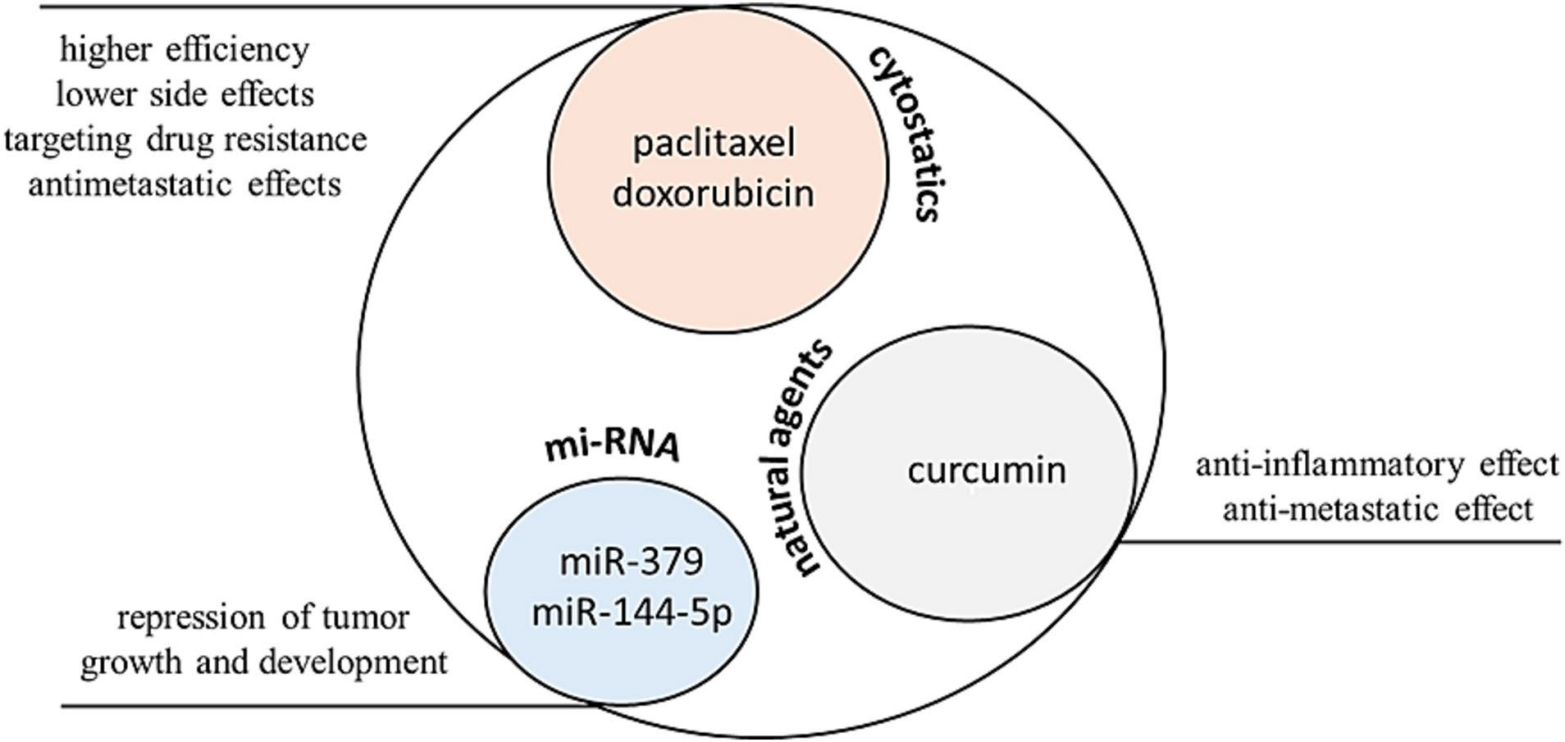
Exosomes represent a promising delivery system for the transport of anticancer agents. In murine cancer models, specifically in breast and ovarian carcinoma, exosomal formulations exhibit lower cardiotoxicity and higher efficacy compared to free doxorubicin. In the case of paclitaxel, exosomal formulations provide significantly improved intracellular delivery into 3LL-M27 cells (Lewis carcinoma expressing P-glycoprotein, which is associated with drug resistance) when compared to liposomal formulations and polystyrene nanoparticles. Furthermore, in these murine models, exosomes have been shown to suppress the development of metastases. Regarding natural agents, curcumin-loaded exosomes display promising efficacy against colon and pancreatic carcinomas, exhibiting antimetastatic effects and reduced inflammation. Exosomes are also suitable for the transport of biological agents such as microRNAs. For instance, exosomes loaded with miR-379 and miR-144-5p demonstrate potent effects against breast cancer and pancreatic ductal adenocarcinoma. The figure was partly generated using Servier Medical Art, provided by Servier, licensed under a Creative Commons Attribution 3.0 unported license.

The two main areas in which exosome applications are greatly investigated and hold great potential are cancer and NDs (74, 76). In cancer treatment, significant attention is paid to nanocarriers which can entrap chemotherapeutic drugs and deliver them to the diseased site, reducing the side effects associated with the systemic administration of conventional anticancer drugs (148, 149). In recent years, exosomes started to be explored as promising nanocarriers (48) that can affect tumor growth, metastasis, and even sensitize cancer cells to conventional therapies (Figure 6).

Their ability to target specific cell types and deliver therapeutic cargos makes them a valuable asset in the fight against malignancies (150–153). For example, exosomes loaded with doxorubicin (ExoDOX) were used in a mouse model of breast and ovarian cancers. It was found that ExoDOX are less cardiotoxic than free DOX, enabling the use of higher concentrations of ExoDOX, thus increasing the efficacy of DOX (154). ExoDOX conjugated with gold nanoparticles (ExoDOX-GNPs) were used *in vitro* with lung cancer cell lines and normal lung fibroblasts. The pH sensitive conjugation bond enables the enhanced rate of drug release under acidic conditions and successful uptake of the ExoDOX-GNPs by the recipient cells. Cell viability assays indicated that ExoDOX-GNPs exhibit preferential cytotoxicity toward cancer cells and have minimal activity on non-cancerous cells (155). Exosomes loaded with paclitaxel (ExoPTX) were developed. The incorporation of paclitaxel into exosomes enhanced drug cytotoxicity more than 50-fold in drug-resistant MDCKMDR1 (Pgp+) cells (156). Furthermore, ExoPTX displayed a potent anticancer effect in a mouse model of murine Lewis Lung Carcinoma pulmonary metastases. In addition to separate therapeutics, other compounds are also used as exosomal cargo. Exosomes loaded with gemcitabine in combination with the survivin protein with mutation T34A induce apoptosis and enhance gemcitabine-killing effects in pancreatic adenocarcinoma cells (157). For cancer treatment, exosomes can also deliver nucleic acids, including miRNAs, siRNAs, and mRNAs (158). For instance, exosomes containing miR-379, a potential tumor suppressor, showed a significant reduction in the rate of tumor formation and growth for the *in vitro* and *in vivo* therapies of breast cancer (159). Exosomes loaded with miR-145-5p, which inhibits pancreatic ductal adenocarcinoma (PDAC) cell proliferation, invasion, and increase apoptosis, significantly reduced the growth of xenograft tumors in a PDAC mouse model (160). Natural products are also used in exosomal cancer therapies, including the use of curcumin. This molecule can mitigate cancer initiation and metastasis (161, 162). Exosomes loaded with curcumin induced the apoptosis of pancreatic cancer cells and significantly delayed brain tumor growth with reduced inflammation when delivered to a GL26 brain tumor model via an intranasal route (163, 164). Plant exosomes with curcumin are used also in Phase I clinical trial investigating the ability of plant exosomes to deliver curcumin to normal and malignant colon tissue (NCT01294072). This study is now recruiting patients (165). Grape exosomes are investigated in preliminary active clinical trial to abrogate oral mucositis induced by combined chemotherapy and radiation in head and neck cancer patients (NCT01668849) (165). Vaccination with tumor antigen-loaded dendritic cell-derived exosomes on patients with unresectable NSCLC lung cancer responding to induction chemotherapy was explored in another clinical study (NCT01159288), where the first phase of this study has now been completed. The primary endpoint was progression-free survival at 4 months after chemotherapy cessation, with a target of at least 50% of patients achieving this endpoint. However, this target was not met as only 32% of patients experienced disease stabilization at 4 months (165, 166). A study of mesenchymal stromal cell-derived exosomes with KrasG12D siRNA for metastatic pancreas cancer patients harboring the KrasG12D mutation is now recruiting patients for the NCT03608631 clinical trial (166). G12D is the most common KRAS mutation detected in carcinomas and confers a unique structural conformation that influences downstream signaling and may lead to its potent oncogenic activity (167).

**Figure 7:**
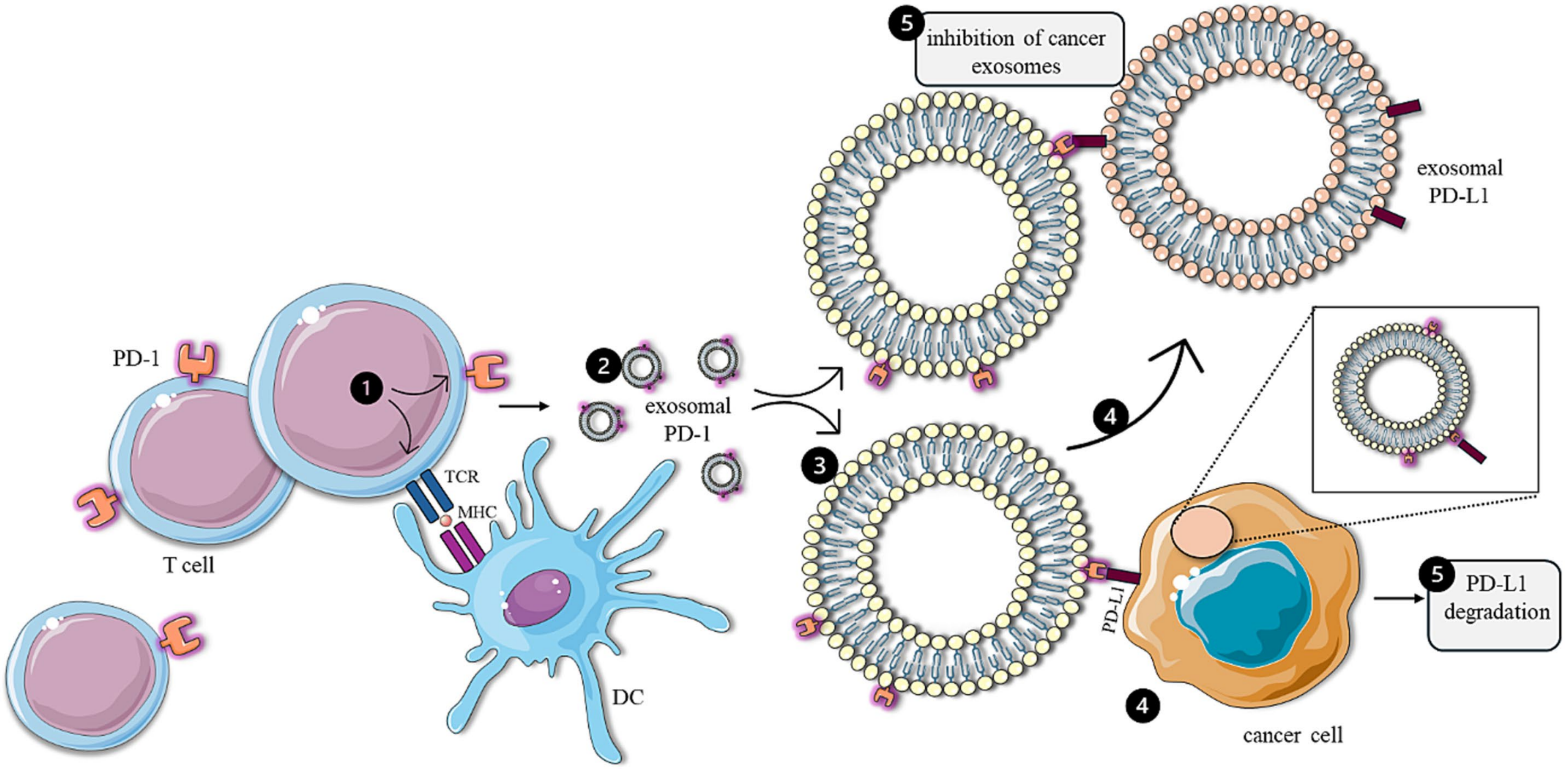
Anti-tumor effects of T-cell-derived exosomes. Following activation by antigen-presenting cells (1), T cells generate exosomes containing PD-1 (2), which directly attenuate the immunosuppressive effects of cancer cells (4) by facilitating the degradation of PD-L1 (5) present on the surface of tumor cells. In addition, T-cell-derived exosomes (3) inhibit the functionality of immunosuppressive exosomes expressing PD-L1 (5), produced by cancer cells. The figure was partly generated using Servier Medical Art, provided by Servier, licensed under a Creative Commons Attribution 3.0 unported license.

It is noteworthy that anti-tumor exosomes could be produced by activated T cells themselves (Figure 7). It is well established that tumor cells often express programmed death-ligand 1 (PD-L1) to deactivate T cells through the activation of programmed death-1 (PD-1) signaling pathways (168). Furthermore, research has shown that these tumor cells also release exosomes containing PD-L1, which exhibit similar immunosuppressive functionality. However, Qiu et al. have reported that activated T cells produce exosomes containing PD-1, which serve to neutralize exosomes carrying PD-L1 (169) and induce the degradation of PD-L1 on the surface of TNBC cells. This dynamic interplay highlights the potential of T-cell-derived exosomes in counteracting tumor-mediated immune evasion.

**Figure 8:**
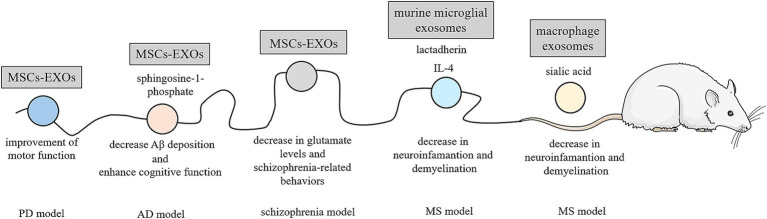
Exosomes in the treatment of neurodegenerative diseases. Due to their ability to cross the BBB, exosomes are being investigated for the treatment of neurodegenerative diseases. In a rat model of PD, bone marrow-derived mesenchymal stem cell exosomes (MSCs-EXOs) have been shown to improve motor function. Similarly, MSCs-EXOs with a high content of sphingosine-1-phosphate have been found to decrease Aβ deposition and enhance cognitive function in a mouse model of AD. In a mouse model of schizophrenia, MSCs-EXOs have been reported to reduce cerebrospinal fluid glutamate levels and alleviate schizophrenia-related behaviors. Exosomes derived from murine microglial cells contain IL-4, an anti-inflammatory cytokine, and lactadherin, a phagocytic “eat me” signal, which reduce neuroinflammation in a mouse model of experimental autoimmune encephalomyelitis (a MS model). Similarly, effects were observed with macrophage-derived exosomes that were modified with derivatives of sialic acid, containing resveratrol. The figure was partly generated using Servier Medical Art, provided by Servier, licensed under a Creative Commons Attribution 3.0 unported license.

The ability of exosomes to cross the blood–brain barrier (BBB) provides the opportunity for their use in the treatment of NDs (45, 170). Many publications highlight the application of MSCs-EXOs in the treatment of NDs (Figure 8) (171, 172).

For example, the effect of bone marrow-derived MSCs-EXOs was examined in rats with induced Parkinson’s disease (PD). Rats treated with these exosomes showed significant improvements in motor function and histopathological outcomes, demonstrating a greater suppression of PD symptoms compared to L-DOPA treatment, which is a medication commonly used to treat PD (173). In AD treatment, bone marrow MSCS-EXOs with high content of sphingosine-1-phosphate (second messenger downregulated in the AD tissue) were injected into double transgenic AD mice (174). Their application led to reduce Aβ deposition and promote cognitive function recovery. According to the observed results, sphingosine kinase inhibitor (SKI-II) or sphingosine-1-phosphate 1 receptor blocker (VPC23019) repress the therapeutic effects of exosomes. Intracerebral injection of bone marrow MSCS-EXOs in a preclinical mouse model of early stage of AD suggests the possibility of intervening before overt clinical manifestations. The study indicated that bone marrow MSC-EXOs are effective at reducing the Aβ plaque burden and the number of dystrophic neurites in both cortex and hippocampus (175). Adipose-MSCs-EXOs administered intranasally and HUCMSCs-EXOs administered intravenously have also shown promise in AD treatment. These treatments effectively improved neurological damage in entire brain regions, increased newborn neurons, powerfully rescued memory deficits, and reduced Aβ expression in transgenic AD mice (176–179). MSCS-EXOs were also used in a mouse model of schizophrenia, where they improved the core schizophrenia-like behavior and biochemical markers of schizophrenia (e.g., cerebrospinal fluid glutamate level) (180). In addition to MSCs-EXOs, macrophage-derived EXOs loaded with resveratrol, nature antioxidant, or exosomes from murine microglia cell line containing anti-inflammatory cytokine IL-4 were also used in mice with MS (181, 182). The efficacy of this approach can be increased by suitable derivatization of the exosome membrane with compounds such as lactadherin (“eat me” signal for the phagocytes) or sialic acid derivatives (BBB transport). Both exosome agents significantly inhibited inflammatory responses in the CNS and the peripheral system in a mouse model and effectively improved the clinical evolution of MS *in vivo* (182, 183). In clinical studies, there is just a single trial registered in CLinicalTrials.gov. This study evaluates the safety and the efficacy of adipose MSC-EXOs in AD (NCT04388982). It was found that the intranasal administration of MSCs-EXOs was safe and well tolerated for a semi-weekly treatment frequency. A dose of at least 4 × 10^8^ particles was selected for a randomized phase II and phase III clinical trial in further steps (181).

## 5 Future direction

Exosomes are increasingly recognized for their pivotal role in the diagnostic realm of cancer and NDs. Nonetheless, a multitude of high-impact clinical trials have unveiled their promising potential in diagnosing a spectrum of additional serious conditions. Hyperuricemia (HUA) is acknowledged as a significant risk factor for chronic heart failure (CHF), a disease frequently linked to elevated morbidity and mortality rates (184, 185). Notably, fluctuations in miRNA expression have been correlated with cardiovascular diseases, including CHF and HUA (186, 187). Analysis of miRNA patterns in serum exosomes revealed that miR-27a-5p was upregulated (*p* < 0.01), while miR-139-3p was downregulated (*p* < 0.01) in patient groups (CHF with HUA) (188). When used in combination, these markers exhibited an AUC of 0.899 (95%) with 79.2% sensitivity and 91.7% specificity. Moreover, exosomes are integral to the pathogenesis of osteoarthritis, with those isolated from synovial fluids emerging as promising diagnostic instruments for affected patients (189, 190). However, exosome isolation methods can be invasive. In contrast, urine biomarkers present a non-invasive alternative for osteoarthritis diagnostics (191), positioning urine-derived exosomes as potential diagnostic assets. Cao et al. introduced a nanopolymer modified with an exosome-affinity component (CD63 aptamer and distearoyl phosphoethanolamine) (192). This innovative approach leads to aggregation of exosomes upon binding, facilitating easy centrifugation (4,000 g for 3 min at pH 6). Notably, these precipitated exosomes can be re-dissolved in a basic pH environment. Metabolomic analyses of urine exosomes have identified 30 biomarkers, including catechol (AUC = 0.917, *p* < 0.001), which effectively differentiate between healthy individuals and those with early osteoarthritis.

In the future, exosomes such as natural nanocarriers should emerge as a novel therapeutic alternative in the fields of oncology, immunology, and regenerative medicine. Future research may focus on refining loading techniques, optimizing targeting strategies, and exploring novel applications in diverse disease contexts (193). For example, in ND treatment to enhance targeted drug delivery efficiency, exosomes can be surface-modified with RVG protein/peptides to specifically bind to the acetylcholine receptor expressed on neuronal cells (194). Comparing RVG-tagged MSC-EXOs to MSCS-EXOs, EXOs tagged with RVG exhibit improved targeting to the cortex and hippocampus after being administered intravenously, plaque deposition and Aβ levels are decreased sharply, and the activation of astrocytes is obviously reduced compared to the observations made in the group of AD mice treated only with MSCs-EXOs. In the group of AD mice injected with RVG-EXOs, there is a significant improvement in learning and memory capabilities with reduced plaque deposition and Aβ levels (195).

Exosomes can also be combined with other biomaterials or inorganic materials for biomedical uses. These hybrid nanoparticles can be further loaded with specific cargo or drug and surface engineered to increase the local concentration of the particles at the diseased site, thereby reducing toxicity and side effects and maximizing therapeutic efficacy (196). The surface engineered exosomal hybrid nanoparticles with specific cargo loading, and modifications conferring desired properties such as pH sensitivity or photosensitivity can be called “ExoBots.” This term reflects the hybrid nature of these structures, which incorporate elements from both biological (exosomes) and technological (robotics) realms to form advanced nanoparticle system. ExoBots combine the advantageous properties of exosomes and other nanoparticles, holding great promise for advancing therapeutic interventions across various biomedical applications. For instance, an engineered core-shell hybrid system was prepared for the *in vivo* treatment of PD mice. This hybrid system consisted of a curcumin-loaded polymer nanoparticle core and an RVG-modified exosome shell. This hybrid was able to clear *α*-synuclein aggregates, reduce their cytotoxicity in neurons, and improve the motor behavior of PD mice (197).

Another type of hybrid is composed from exosomes and liposomes. Long circulating and pH sensitive hybrids loaded with DOX were investigated for anti-tumor effect on a mouse model of breast cancer. The results indicated that this hybrid system may be a promising nanocarrier for the treatment of breast cancer, reducing toxicity and inhibiting metastasis mainly in the lungs (198). This hybrid approach was also used to overcome chemotherapy resistance in OC. The hybrid system was developed by fusing cRGD-modified liposomes loaded with miR-497 and triptolide with CD47-expressing tumor-derived exosomes. RGD peptides specifically bind to integrin receptors overexpressed on the surface of many cancer cells. Overexpression of miR-497 may overcome OC chemotherapy resistance, and triptolide was confirmed to possess a superior killing effect on cisplatin-resistant cell lines. The *in vitro* results indicated that these hybrids were efficiently taken up by tumor cells, thus significantly enhancing tumor cell apoptosis and exerting significant anticancer activity without any negative effects observed *in vivo*. These hybrids may provide a translational strategy to overcome cisplatin-resistant OC (199). In addition, paclitaxel was used as the cargo of hybrids for *in vivo* colon cancer treatment. The study revealed that hybrids significantly suppressed tumor growth in a colon tumor-bearing mouse model, reduced the expression of M2 type tumor-associated macrophages, and decreased regulatory T cells (200). Many other types of hybrids were explored, such as thermosensitive hybrids for improved treatment of metastatic peritoneal cancer, pH sensitive macrophage hybrids loaded with DOX for tumor targeted drug delivery, or long circulated pH sensitive hybrids loaded with dasatinib for pancreatic cancer treatment. All these ExoBots show positive therapeutic results and can thus serve as potential therapeutics for cancer treatment (201–203).

Recently, alongside the extensive research on exosomes derived from eukaryotic cells, there has been a growing interest in exploring plant-derived exosomes (P-ELNs; puerarin) as a novel source of exosomes with potential applications in various biotechnological and therapeutic fields. Polyphenolic compounds found in various plant exosomes show great promise in treating serious health disturbances. High-impact studies (204, 205) suggest that their therapeutic effects may be partially attributed to their ability to target ferroptosis, a process linked to numerous pathological states (206, 207).

For instance, exosome-like nanovesicles derived from *P. lobata* roots have been shown to alleviate alcoholic intoxication, enhance alcohol metabolism, and reduce alcohol levels in the liver and serum of mouse models (204). These effects are associated with the induction of acetaldehyde dehydrogenase activity and a decrease in glutathione peroxidase 4 and glutathione levels, as well as with the suppression of acyl-CoA synthetase long-chain family member 4, likely contributing to the repression of ferroptosis. In addition, *Robinia pseudoacacia L*. flower-derived P-ELNs, when administered orally, significantly reduce hypoxia-induced ferroptosis and mucosal injury in the gastrointestinal tract of mouse models (205). Their effects are mediated through the modulation of HIF-1α and HIF-2α expression, subsequently influencing ROS production and lipid peroxidation via NOX4 and ALOX5 pathways.

Another promising application of P-ELNs is their potential to mitigate obesity, a well-known risk factor for cancer, metabolic disorders, cardiovascular diseases, and inflammation (208–210). The global prevalence of obesity has been on the rise, making this research particularly relevant. Wang et al. reported that turmeric-derived ELNs exhibit potent anti-obesity effects, achieving weight reductions of 8.68 and 14.56% through intragastric and subcutaneous delivery, respectively (211). This effect is linked to the stimulation of adipocyte apoptosis, the induction of lipolysis, and the inhibition of lipogenesis, highlighting the therapeutic potential of these natural compounds. However, the biological functions of P-ELNs are not fully understood, standard isolation protocols are lacking, and P-ELNs are a promising new frontier in precision medicine. Their plant origin offers advantages in terms of biocompatibility, scalability, and reduced immunogenicity. Moreover, bioactive molecules from plants are associated with disease-preventive effects making P-ELNs an attractive alternative to mammalian EVs in future biomedical innovations (212–214).

## 6 Conclusion

Although further fundamental research, especially regarding the biogenesis of exosomes and the optimization of their isolation techniques and characterization methods, is necessary, their significant potential has been demonstrated in several biomedical areas, particularly in regenerative medicine, disease diagnosis, and treatment. The continued growth of the exosome market is also evidenced by the recent announcement of four collaborations between pharmaceutical companies, including two potentially worth close to $1 billion each. One of the largest is Lilly’s partnership with Evox Therapeutics of Oxford, UK. In this deal, which could bring in up to $1.2 billion in milestone payments, CNS-targeting exosomes developed by Evox will be loaded with RNA interference and antisense oligonucleotide therapies from Lilly, targeting up to five undisclosed targets. In another major deal, potentially worth over $900 million, Carmine Therapeutics has partnered with Takeda to develop gene therapies for two undisclosed rare diseases targets (215). Exosomes may thus be the future of medicine, used as ExoBots programmed to deliver specific drugs to specific locations within the organism with minimal side effects and high therapeutic efficacies.

## Glossary

AD: Alzheimer’s disease
ALS: amyotrophic lateral sclerosis
BBB: blood–brain barrier
CHF: chronic heart failure
DGUC: density gradient ultracentrifugation
DLS: dynamic light scattering
DUC: differential ultracentrifugation
EEs: early endosomes
EGFR: epidermal growth factor receptor
ELISA: enzyme-linked immunosorbent assay
EMT: epithelial–mesenchymal transition
EVs: extracellular vehicles
ExoDOX: exosomes loaded with doxorubicin
ExoDOX-GNPs: exosomes loaded with doxorubicin conjugated with gold nanoparticles
Exo-L: large exosomes
ExoPTX: exosomes loaded with paclitaxel
Exo-S: small exosomes
HUA: hyperuricemia
HUCMSCs-EXOs: mesenchymal stem cell exosomes derived from human umbilical cord
ILVs: intraluminal vesicles
ISEV: International Society for Extracellular Vesicles
LC–MS: liquid chromatography–mass spectrometry tandem technique
LEs: late endosomes
MS: multiple sclerosis
MSCs: mesenchymal stem cells
MSCs-EXOs: mesenchymal stem cell-derived exosomes
MVBs: multi-vesicular bodies
nanoFCM: nanoflow cytometry
NDs: neurodegenerative diseases
NGS: next-generation sequencing
NSCLC: non-small cell lung cancer
NTA: nanoparticle tracking analysis
PD: Parkinson disease
PDAC: pancreatic ductal adenocarcinoma
PD-1: programmed death-1
PD-L1: programmed death-ligand 1
P-ELNs: plant-derived exosomes
qRT-PCR: quantitative reverse transcription polymerase chain reaction
RPS: resistive pulse sensing
SEC: size exclusion chromatography
SERS: surface-enhanced Raman spectroscopy
TEXs: tumor-derived exosomes
TFF: tangential flow filtration
TME: tumor microenvironment
TNBC: triple-negative breast cancer
OA: osteoarthritis
UF: ultrafiltration
