# Supplementary material for: The potential of exosomes in regenerative medicine and in the diagnosis and therapies of neurodegenerative diseases and cancer
Source: Front Med (Lausanne). 2025 Mar 13;12:1539714. doi: 10.3389/fmed.2025.1539714 (PMC11966052; doi:10.3389/fmed.2025.1539714)
Supplement: Supplementary file 1 [file Table_1.docx]

Table S1: Exosomes biomarkers in cancer disagnosis

| **Marker** | **Source** | **Exosomes characterization** | **Finding** | **Ref.** |
| --- | --- | --- | --- | --- |
| **Lung cancer** | | | | |
| CD151 (TSPAN24) | 431 patients with lung cancer vs 150 controls | Plasma  30 - 100 nm  antibody^[[1]](#footnote-1)^ (CD9, CD81, and CD63) | **↑**(patients vs control, AUC = 0.68, p = 0.0002) | [1] |
| Tetraspamin 8 |  |  | **↑**(patients vs control, AUC = 0.60, p = 0.0002) |  |
| CD171 |  |  | **↑**(patients vs control, AUC = 0.60 p = 0.0002) |  |
| PD-L1 | 17 locally advanced or metastatic NSCLC patients | Serum  ~100nm  marker^[[2]](#footnote-2)^ (CD63, CD81 and HSP70) | **↑**(patients vs control); **↑**(tumour progression) | [2] |
| PD-L2 |  |  | **↑**(patients vs control) |  |
| PD-1 |  |  | **↑**(patients vs control); **↑**(response in ICS therapy, p<0.0156) |  |
| **Breast cancer** | | | | |
| GLUT-1 | MDA-MB-231 vs  MCF-10A cells | Exosome free medium  ~105 nm  marker (CD63 and CD81) | Selective expression in cancer cell line exosomes | [3] |
| GPC-1 |  |  |  |  |
| ADAM10 |  |  |  |  |
|  | MDA-MB-468 cells after EGFRi treatment | conditioned medium  ~180 nm  TSG101 and HSP70 (positive and negative markers, respectively) | Resistance exosomal EGFR against autophosphorylation inhibition;  **↑**cell proliferation | [4] |
| miR-1246 | 16 breast cancer patients vs 16 control | Plasma | **↑**(patients vs control) | [5] |
| miR-21 |  |  |  |  |
| miR-1246 | 46 breast cancer patients vs 28 controls | Plasma  Au Nanoflare Probe | **↑**(patients vs control, AUC = 0.9821, p < 0.0001, 100% sensitivity, 92.9% specificity) | [6] |
| HSP70 | MCF-7 cells | medium depleted from serum-derived exosomes | detection | [7] |
|  | 3 breast cancer patients vs control | urine | **↑**(patients vs control, p < 0.004) |  |
| **Pancreatic cancer** | | | | |
| GPC1 | 190 PDAC pacients vs and vs 100 controls | Serum  ~105 nm  Antibody (GPC1) | **↑**(pancreas cancer vs benign pancreas and control, AUC~1 ; ~100% specifity and sensitivity); GPC1+ crExos level^[[3]](#footnote-3)^: distant metastasis (58.5%), lymph mode metastasis (50.5%), no metastasis (39.9%) | [8] |
| miR-6855-5p | Pancreatic cancer 28 patients (good vs poor radiotherapy response) | peripheral plasma  35 nm size exclusion column | **↓**CA19-9 reduction rate(r = −0.5964, p = 0.0008) | [9] |
| **Colorectal cancer** | | | | |
| CD59 | 73 CRC patients vs 80 control | Peripheral plasma | **↑** (patients vs control, 0.87 AUC);  **↑**distant metastasis (p = 0.0475) | [10] |
| Tetraspanin 9 |  |  | **↑**(patients vs control, 0.87 AUC);**↑**(lymph node metastasis, p = 0.0011); **↑**(distant metastasis, p = 0.0104); **↑**(TNM stage, p = 0.0065). |  |
| ADAM10 |  |  | **↑** (patients vs control, 0.83 AUC) |  |
| Panel (CD59 and Tetraspanin 9) |  |  | **↑**(I/II CRC patients vs controls, 0.98/0.99 AUC) |  |
| **Prostate cancer** | | | | |
| TPP1, | 13 prostatic cancer patients (bladder vs ureter derived) | urinary  antibody (CD9, CD63 and CD81) | **↑**urinary exosomes (bladder derived vs ureter derived) | [11] |
| TMPRSS2 |  |  |  |  |
| FOLFR1 |  |  |  |  |
| TM256 | 17 prostate cancer patients vs 15 control | Urinary  ~149 nm  antibody (CD63)  marker (CD9, Tsg101 and CD81) | 94% sensitivity | [12] |

AUC, area under curve; ADAM10, A Disintegrin and metalloproteinase domain-containing protein 10; CRC, colorectal cancer; EGFR, epidermal growth factor receptor; FOLR1, folate receptor 1; GPC1, glypican-1; GLUT-1, glucose transporter-1; HSP70, heat shock 70 ; MCF-7, breast cancer cell line; MCF-10A, a non-cancerous epithelial breast cell line; MDA-MB-231, triple negative breast cancer cell line; MDA-MB-468, triple negative breast cancer cell line; NSCLC, Non-small cell lung cancer; PDAC, pancreatic ductal adenocarcinoma; TMEM256, (Transmembrane Protein 256; TMPRSS2, Transmembrane protease serine 2; TPP1, Tripeptidyl Peptidase 1; TSG101, Tumor Susceptibility 101

**↑** = positive correlation; **↓**= negative correlation

1. Sandfeld-Paulsen, B., et al., *Exosomal Proteins as Diagnostic Biomarkers in Lung Cancer.* Journal of Thoracic Oncology, 2016. **11**(10): p. 1701-1710.

2. Akbar, S., et al., *Circulating exosomal immuno-oncological checkpoints and cytokines are potential biomarkers to monitor tumor response to anti-PD-1/PD-L1 therapy in non-small cell lung cancer patients.* Front Immunol, 2022. **13**: p. 1097117.

3. Risha, Y., et al., *The proteomic analysis of breast cell line exosomes reveals disease patterns and potential biomarkers.* Scientific Reports, 2020. **10**(1): p. 13572.

4. Hung, Y., et al., *The exosomal compartment protects epidermal growth factor receptor from small molecule inhibitors.* Biochemical and Biophysical Research Communications, 2019. **510**(1): p. 42-47.

5. Hannafon, B.N., et al., *Plasma exosome microRNAs are indicative of breast cancer.* Breast Cancer Research, 2016. **18**(1): p. 90.

6. Zhai, L.-Y., et al., *In Situ Detection of Plasma Exosomal MicroRNA-1246 for Breast Cancer Diagnostics by a Au Nanoflare Probe.* ACS Applied Materials & Interfaces, 2018. **10**(46): p. 39478-39486.

7. Gobbo, J., et al., *Restoring Anticancer Immune Response by Targeting Tumor-Derived Exosomes With a HSP70 Peptide Aptamer.* JNCI: Journal of the National Cancer Institute, 2015. **108**(3).

8. Melo, S.A., et al., *Glypican-1 identifies cancer exosomes and detects early pancreatic cancer.* Nature, 2015. **523**(7559): p. 177-82.

9. Ueda, H., et al., *miR-6855-5p Enhances Radioresistance and Promotes Migration of Pancreatic Cancer by Inducing Epithelial-Mesenchymal Transition via Suppressing FOXA1: Potential of Plasma Exosomal miR-6855-5p as an Indicator of Radiosensitivity in Patients with Pancreatic Cancer.* Ann Surg Oncol, 2024.

10. Dash, S., et al., *Extracellular Vesicle Membrane Protein Profiling and Targeted Mass Spectrometry Unveil CD59 and Tetraspanin 9 as Novel Plasma Biomarkers for Detection of Colorectal Cancer.* Cancers (Basel), 2022. **15**(1).

11. Hiltbrunner, S., et al., *Urinary Exosomes from Bladder Cancer Patients Show a Residual Cancer Phenotype despite Complete Pathological Downstaging.* Scientific Reports, 2020. **10**(1): p. 5960.

12. Øverbye, A., et al., *Identification of prostate cancer biomarkers in urinary exosomes.* Oncotarget, 2015. **6**(30): p. 30357-76.

1. Antibody used in the exosome izolations [↑](#footnote-ref-1)
2. Marker observed in the exosomes, [↑](#footnote-ref-2)
3. Ratio of GPC1 positive exosomes in the sample [↑](#footnote-ref-3)
